# Topographic Variation in Human Neurotransmitter Receptor Densities Explains Differences in Intracranial EEG Spectra

**DOI:** 10.1002/hbm.70393

**Published:** 2025-10-31

**Authors:** U. M. Stoof, K. J. Friston, M. Tisdall, G. K. Cooray, R. E. Rosch

**Affiliations:** ^1^ Wellcome Trust Centre for Neuroimaging, UCL Queen Square Institute of Neurology University College London London UK; ^2^ Developmental Neurosciences, UCL Great Ormond Street Institute of Child Health University College London London UK; ^3^ Department of Neurosurgery Great Ormond Street Hospital for Children NHS Foundation Trust London UK; ^4^ Clinical Neuroscience, Karolinska Institutet Stockholm Sweden; ^5^ Departments of Neurology and Paediatrics Columbia University Irving Medical Center New York USA; ^6^ Department of Basic and Clinical Neuroscience Institute of Psychiatry, Psychology and Neuroscience, King's College London London UK

**Keywords:** canonical microcircuit, computational neuroscience, dynamic causal modelling, multimodal model, neural mass model, neurotransmitter

## Abstract

Brain function and its failures arise from dynamical patterns of neuronal activity shaped by synaptic neurotransmission. Both neurotransmitter receptor expression and neuronal population dynamics show a remarkable regional variability across the human cortex. We leverage this functional specialisation to characterise the relationship between receptor architectonics and electrophysiological signals. Using dynamic causal modelling (DCM), we fitted neural mass models to a normative set of intracranial EEG data. Subsequently, Bayesian model comparison helped to evaluate whether models improved when equipped with constraints on synaptic connectivity, based on regional neurotransmitter receptor densities. The results show that dynamic causal models generated region‐specific intracranial EEG spectra accurately. Incorporating prior information on normative receptor distributions further improved model evidence, indicating that regional variation in receptor density explains variations in synaptic connectivity and ensuing cortical population dynamics. The output is a cortical atlas of neurobiologically informed intracortical synaptic connectivity parameters. These can serve as empirical priors in future, patient‐specific models. In summary, we show that molecular cortical characteristics—that is, receptor densities—enrich and inform generative, biophysically plausible models of coupled neuronal populations. This work helps to explain regional variations in human electrophysiology, provides a methodological foundation to integrate multimodal data, and serves as a normative resource for future DCM studies of electrophysiology.

## Graphical abstract

Using generative models, we demonstrated that oscillatory neuronal population activity is shaped by the distribution of neurotransmitter receptors. This modelling furnishes an atlas of normative parameter values, which constitute neurobiologically informed priors for in silico (e.g., Digital Twin) characterisation of normal and disordered brain functioning.

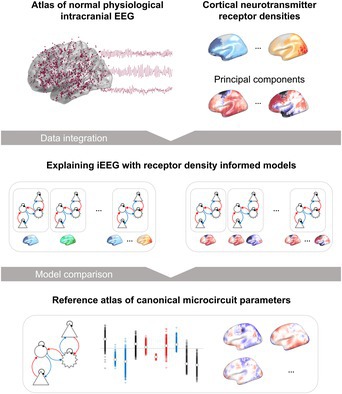

## 1 Introduction

Translation between spatial neural scales is a central challenge in neuroscience, for understanding neurological disorders and for leveraging molecular and cellular insights in clinical applications. Mechanistic descriptions of pathologies, often formulated at the level of synapses and cells (Akyuz et al. 2021; Bennett 2000; Malhi and Mann 2018; Owen et al. 2016), differ in spatial scale from measurements of human brain function, such as intracranial and scalp electroencephalography (i/EEG) or functional magnetic resonance imaging (fMRI), which are used in guiding treatments of human brain disorders such as epilepsy (Bartolomei et al. 2017; Müller et al. 2018; Zijlmans et al. 2019). The difficulty in integrating varying levels of description lies in the structural and functional complexity of the brain, which is characterised by interrelated sub‐systems and their non‐trivial relationship to emergent regional and whole brain functions, and the fact that meso‐ and macroscale dynamics shape and constrain the activity of the constituent microscale components (“circular causality”) (Braun 2016; Freeman 2000, 2007a, 2007b; Friston 2019; Haken 1984; Hoel et al. 2013; Turkheimer et al. 2022).

To illustrate: many aspects of synaptic mechanisms underlying neuron‐to‐neuron interaction have been revealed both in healthy brains and in disorders (Akyuz et al. 2021; Amzica and da Lopes Silva 2017; Malhi and Mann 2018; Owen et al. 2016; Südhof 2018; Südhof and Malenka 2008), but how cellular interactions and entangled neurotransmitter systems influence neuronal population activity represents a challenging problem of emergence (Marder 2012; Næss et al. 2021; Shine et al. 2019, 2021). Conversely, phenomena of human brain (dys)function are described at macroscopic scales and measured with tools such as EEG (Beal et al. 2017; de Aguiar Neto and Rosa 2019; Krishnan et al. 2017; McMackin et al. 2019; Perrottelli et al. 2021), but deconstructing these signals to unravel how the underlying interacting neurotransmitter systems contribute, is an ill‐defined inverse problem (Breakspear 2017).

Addressing this circular explanatory gap has important practical implications. For example, neuropharmacological interventions, such as anticonvulsants or antidepressants, rely on interpreting clinical phenotypes to target specific mechanisms or components of neurotransmission and neuromodulation. And whilst medications' affinity for types of neurotransmitter receptors is often well characterized at microscale (McKarns 2023), their effects on meso‐ and macroscopic dynamics are less accessible—as they depend on a multitude of factors, including balancing mechanisms of non‐targeted interacting neurotransmitter systems (Luppi et al. 2023). Conversely, measuring and describing patients' brain dysfunction, allows only phenomenological, but not aetiological, diagnoses (Fisher et al. 2017; Scheffer et al. 2017). In consequence, the disconnect between levels of description impedes progress towards individualised patient care (Jafarian et al. 2023; Kringelbach et al. 2020; Lawn et al. 2023; Luppi et al. 2022).

Despite the complexity, there is evidence that interrelations between synaptic neurotransmitter systems and whole‐brain dynamics are identifiable. For example, there are statistical associations between regional cortical oscillatory rhythms measured using magnetoencephalography (MEG) and neurotransmitter receptor expression measured using positron emission tomography (PET) (Hansen et al. 2022), and between multiple microstructural features of the human cortex and MEG signals (Shafiei et al. 2023); additionally, regional neuroreceptor profiles in part explain medication‐induced changes in fMRI (Luppi et al. 2023). Similarly, spatially distributed neuroreceptor gene expression, assessed through post‐mortem microarray profiles, correlates with fMRI activation patterns during cognitive tasks along gradients of microstructural organisation of the human cortex (Hansen et al. 2021).

This illustrates that statistical relationships between neurotransmitter receptor compositions and cortical rhythms emerge at the level of brain regions. At that spatial scale, neurotransmitter receptor densities (RD) might serve as proxies for neuronal functioning (Goulas et al. 2021; Zachlod et al. 2023; Zilles and Palomero‐Gallagher 2017). For example, glutamatergic AMPA and NMDA, and GABAergic GABA_A_ RD vary largely across the cortex (Zilles and Palomero‐Gallagher 2017) and show pronounced alterations in pathological tissue (Palomero‐Gallagher et al. 2012). Consequently, these normative, post‐mortem, in vitro autoradiography data of distinct receptors might inform multimodal in silico methods aiming to uncover how receptors contribute to brain dynamics, and positive findings in previous statistical (Hansen et al. 2022) and disease pathway models (Khan et al. 2022, 2023) motivate our investigation.

Obtaining accurate measurements of neuronal population dynamics (electrophysiological data) with non‐invasive methods is challenging (Glomb et al. 2022; Nunez and Srinivasan 2005). Alternatively, intracranial EEG—which is now routinely used in epilepsy presurgical assessments to identify pathological networks (Bartolomei et al. 2017; Müller et al. 2018; Thornton et al. 2011; Zijlmans et al. 2019)—captures local activity with high temporal resolution and signal‐to‐noise ratio (Parvizi and Kastner 2018). Similar to neuroreceptor densities, iEEG signals are region specific: this was revealed by an atlas of putatively normal intracranial recordings remote from epileptogenic tissue, which showed regionally characteristic iEEG power spectral densities (PSD) in canonical frequency bands (Frauscher et al. 2018b). Therefore, intracranial EEG offers precise measurements of regional brain activity, explainable by the microarchitecture and functioning of neuronal populations.

To investigate the putative neurobiological mechanisms underlying electrophysiological signals such as i/EEG, computational models of coupled neuronal populations have been widely applied to address a range of neuroscientific questions (Breakspear 2017; Deco et al. 2008; Destexhe and Sejnowski 2009; Doelling and Assaneo 2021; Glomb et al. 2022; Wendling and da Lopes Silva 2017). But, although the parameters of these generative models are inspired by the biophysical properties of neuronal populations, relating these to the complex interacting neurotransmitter systems in the human brain has remained challenging.

A possible computational approach to integrate both iEEG and neuroreceptor data and to thereby provide neurobiological grounding, is to incorporate the receptor features as prior information to model fitting of electrophysiological recordings. Bayesian approaches such as dynamic causal modelling (DCM) (Bastos et al. 2012; David et al. 2006; Friston et al. 2003; Kiebel et al. 2006, 2008; Moran et al. 2009, 2013; Wang and Liang 2021) allow for this integration of empirically derived priors (Friston et al. 2016, 2015), which enables us to evaluate different hypotheses about how neuroreceptor composition contributes to regional oscillatory signatures (iEEG).

In summary, we used DCM—as a computationally efficient method—to link recordings of regional brain dynamics and neuroreceptor densities through empirically informed generative models of neuronal population function. Through this approach, we provide evidence that neuronal oscillations in the human brain are shaped by regional variation in neuroreceptor densities and, as an output, we generated an atlas of neuronal population parameters for the canonical microcircuit (CMC) model that can inform future DCM studies.

## 2 Methods

### 2.1 Overview

**FIGURE 1:**
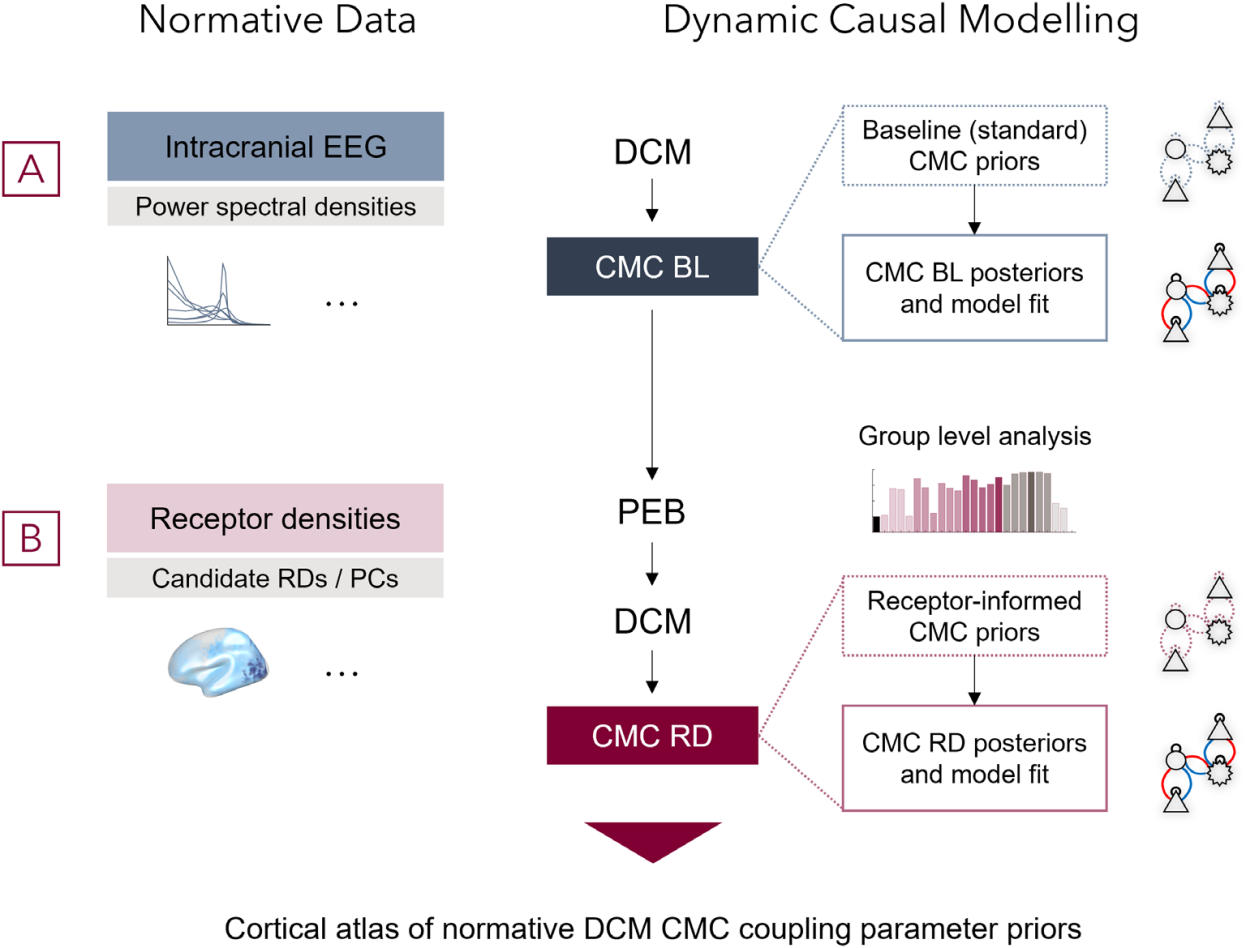
Modelling approach to integrate cortical dynamics and neuroreceptor density maps. (A) Interictal iEEG traces without overt pathological appearances were previously collated across 106 patients and 1770 cortical contacts. Trace characteristics were summarised as power spectral densities (PSD) and individual four population models (canonical microcircuit model [CMC]) of cortical dynamics were fit to each individual contact's trace using a variational Bayesian model fitting approach (dynamic causal modelling [DCM]) resulting in CMC baseline models (CMC BL) with generated PSDs and parameter estimates. (B) Density profiles of 15 neurotransmitter receptors (Table S1) in 44 cortical regions were incorporated as covariates in a hierarchical analysis with parametric empirical Bayes (PEB) to assess if and how different combinations of receptors improve evidence. Two sets of hypotheses were tested: (a) including only four major GABAergic (GABA_A_, GABA_B_) and glutamatergic (AMPA, NMDA) receptors, (b) capturing regional variation of all receptors with principal component analysis (PCA) and using these components as covariates. The evidence of all receptor density (RD) informed models was compared. For the winning model, three sets of randomised null models were used to validate results, and its priors constitute the normative cortical parameter atlas for electrophysiological DCM CMCs.

For this study, two sets of data and computational approaches were used (Figure 1). First, to model power spectral densities for previously collected intracranial EEG data (Frauscher et al. 2018b), we employed an established neural mass model, the canonical microcircuit (Bastos et al. 2012; Pinotsis et al. 2013) (Figure 1A), and inferred its parameters using DCM (David et al. 2006; Friston et al. 2003; Kiebel et al. 2006, 2008; Moran et al. 2009). Subsequently, in a second‐level analysis with parametric empirical Bayes (Friston et al. 2016, 2015; Zeidman et al. 2019) we tested which combinations of regional neurotransmitter receptor densities (Zilles and Palomero‐Gallagher 2017) (Figure 1B, Supplementary Table S1) improve model evidence (i.e., variational free energy), across all individually fitted regional CMC models.

### 2.2 Normal Physiological Intracranial EEG Data

**FIGURE 2:**
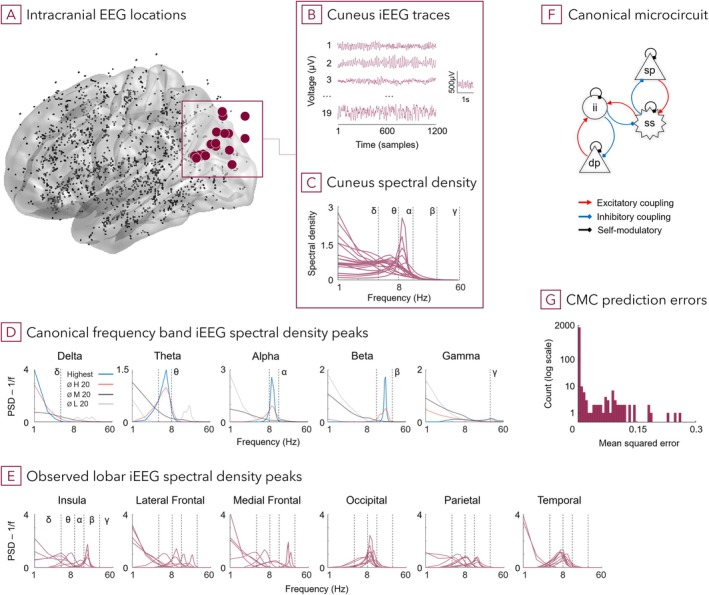
Cortical microcircuit models explain a diversity of regionally specific cortical iEEG patterns. (A) iEEG contacts, projected onto a MNI ICBM152 standard single hemisphere pial surface. 1770 traces were included, obtained from 106 patients under awake, eyes‐closed conditions without visually apparent pathological features. Red dots highlight cuneus contacts illustrated in subsequent panels. Contacts which appear to be outside the restrictive pial surface boundary still carried valid signals and were included in the regional mapping by Frauscher et al. (2018b). (B) Example 5‐second traces recorded from cuneus contacts. (C) Cuneus' observed iEEG‐derived PSDs (original data, individual contacts). (D) Examples of periodic (oscillatory) components—iEEG power spectral density (PSD) minus aperiodic component (1/frequency)—in canonical frequency bands (delta (*δ*, 1–4 Hz), theta (*θ*, 4–8 Hz), alpha (*α*, 8–13 Hz), beta (*β*, 13–30 Hz), and gamma (*γ*, 30–60 Hz)). Blue lines indicate the single iEEG trace with the greatest spectral power—relative to 1/f noise—within the respective frequency band; red/black/light grey lines are means of the 20 highest/middle/lowest iEEG PSDs within a band. For frequencies where the iEEG power < 1/*f*, values were set to 0. (E) Lobar iEEG spectral densities, showing the 10 most significant regional PSDs across the 1–60 Hz frequency range. (F) The canonical microcircuit model with four neural populations (sp—superficial pyramidal, ss—spiney stellate, ii—inhibitory interneurons, dp—deep pyramidal cells), which approximate cortical laminar‐specific activity, was used to model iEEG in the frequency domain. Fourteen posteriors for excitatory (3 parameters) and inhibitory (3) coupling, self‐modulatory (4) and time constant (4) parameters were inverted with variational Bayes under the Laplace assumption (DCM). (G) Most CMC generated PSDs show a good fit and low mean squared error compared with the observation PSDs; a fraction of models (42/1770, MSE > 0.05) had noticeable deviations, which appear primarily in lower frequencies (see Figures S4–S6 for illustrative details).

The neurophysiological dataset comprised 1770 artifact‐free single‐channel traces of iEEG time series (1512 from SEEG recordings, 258 from subdural grids/strips), recorded during 60 s of wakefulness with closed eyes (Frauscher et al. 2018b) (Figure 2A). These recordings were obtained from 106 adult patients with drug‐refractory epilepsy (54/52 male/female, mean age: 33.1 ± 10.8 years) who underwent presurgical evaluation. Electrode contact locations were registered to a normative standard space MNI ICBM152 (Fonov et al. 2011; Mazziotta et al. 2001) with 38 regions, and on average, coverage was 0.9 channels/cm^2^ cortical surface, and 2.7 channels/cm^3^ cortical grey matter.

For this first normative atlas of iEEG (Frauscher et al. 2018b), strict inclusion criteria relating to tissue type (MRI confirmed healthy tissue), location (peri‐implantation imaging with CT/MRI), recording categorisation (additional control iEEG), conditions (standardized, resting wakefulness EEG with eyes closed), and sampling rate (minimum 200 Hz) were applied. The iEEG recordings showed lobe‐, and partially region‐specific PSD with no statistically significant differences between power spectra of homologous regions in cerebral hemispheres. Unmodified iEEG data as obtained from the website were used (Frauscher et al. 2018a). Note, some contacts, which appear to be outside the restrictive pial surface boundary, were included in the original iEEG atlas as they carried valid signals (Figure 2A).

### 2.3 Neurotransmitter Receptor Density Autoradiography Data

**FIGURE 3:**
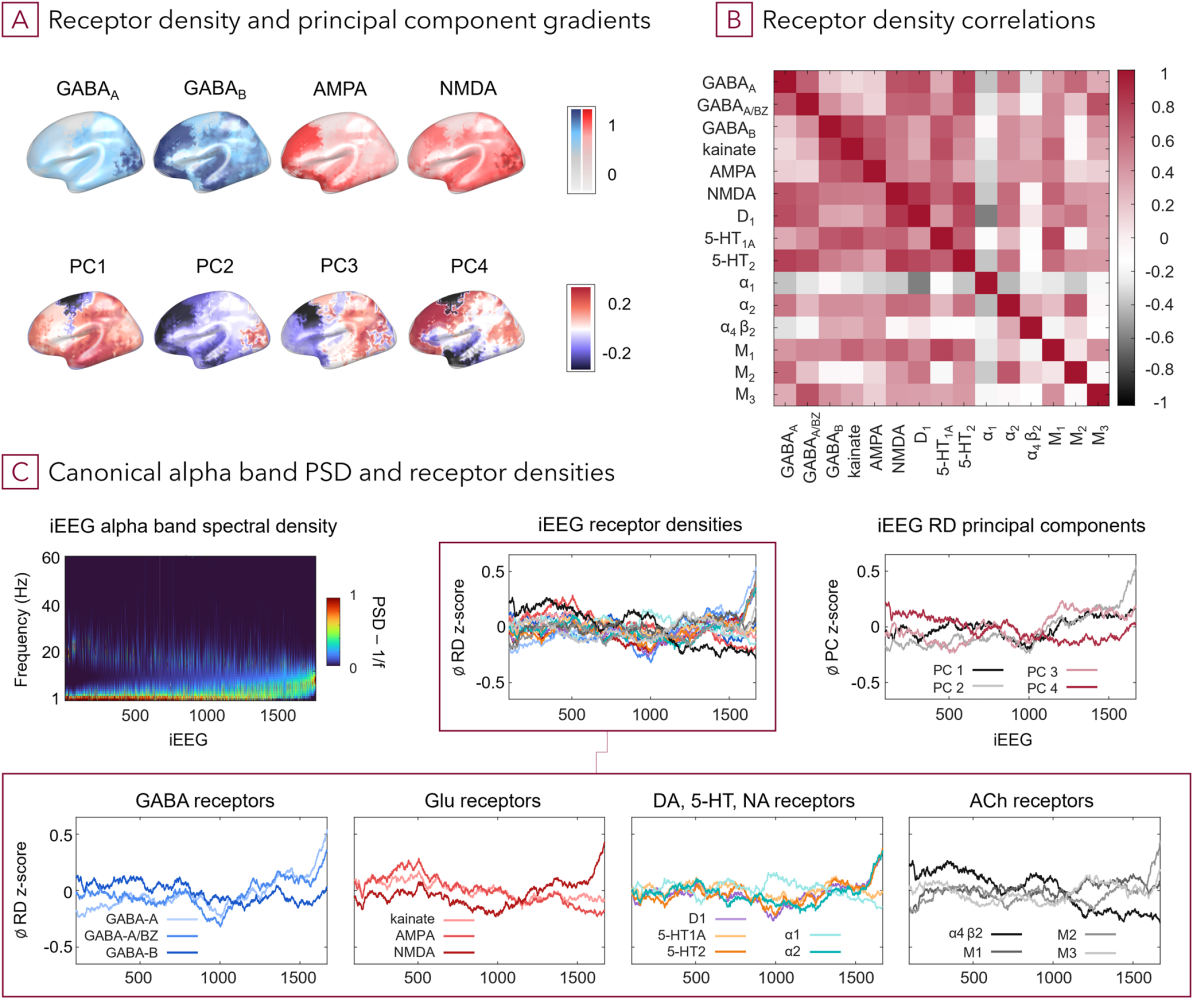
Correlation between receptor densities provides basis for PCA, and trends in specific receptor variation and PSD overlap. (A) Example spatial gradient maps of four neuroreceptor densities and of the first four PCs interpolated from 44 regional averages. (B) Matrix of Pearson's linear correlation coefficients between receptor densities across regions. (C) Relationship between iEEG alpha band spectral power and receptor densities at the iEEG contact location/region. The iEEG channels are sorted in ascending order of increasing periodic alpha spectral power; data of receptor densities and PCs are sorted in the same order. A simple moving average with a window size of 200 was used to smooth RDs' and PCs' *z*‐scores and to capture trends. Details for all frequency bands are shown in Figure S2. Neurotransmitter: Glu—glutamate, DA—dopamine, 5‐HT—serotonin, NA—noradrenaline, ACh—acetylcholine.

Layer‐specific density data of 15 neurotransmitter receptors (Zilles and Palomero‐Gallagher 2017) were available for 44 regions (Amunts and Zilles 2015; Brodmann 1909) (Figure 3A,B). The data were obtained using an in vitro receptor autoradiography protocol (Palomero‐Gallagher and Zilles 2018) on three neurologically healthy donor brains (2/1 male/female, 72–77 age range) (Zilles and Palomero‐Gallagher 2017). Region‐specific balances of RD (Zilles et al. 2002) were estimated, showing, for example, that glutamate and GABA RD vary substantially between cortical regions (Zilles and Palomero‐Gallagher 2017) (Figure S8).

Since RD correlations between layers (supra‐, infra‐, granular) were on average *r* = 0.83 (80% percentile range: [0.62, 0.99]) across receptors, we used normalised unweighted averages of the three layers as regressors in our PEB analyses.

### 2.4 Mapping iEEG and Receptor Density Data

We mapped unihemispheric receptor density sample locations onto the MNI space of the iEEG data. Subsequently, the iEEG contact locations were projected onto the left hemisphere—into the same space as the receptor density data—as no statistically significant hemispheric differences were previously reported for either dataset (Frauscher et al. 2018b; Wagstyl et al. 2020).

### 2.5 Dynamic Causal Modelling

Dynamic causal modelling (David et al. 2006; Friston et al. 2003; Kiebel et al. 2006, 2008; Moran et al. 2009) is a computationally efficient procedure for fitting generative models to empirical timeseries. DCM uses variational Bayes under the Laplace assumption to optimise a variational free energy F (Neal and Hinton 1998) bound on log model evidence $\log p\left(y|m\right)$ (Friston et al. 2007). The objective of this procedure is to find the best approximation $q\left(\theta \right)$ to the true posterior density $p\left(\theta |y,m\right)$ by maximising free energy. Here, y indicates data and m a model with parameters θ.

(1) $$F=\underset{\;\text{evidence}-\text{divergence}}{\log p\left(y|m\right)-D_{\mathrm{KL}}\left[q\left(\theta \right)\|p\left(\theta |y,m\right)\right]}$$

(2) $$\begin{array}{c} F=\underset{\;\text{accuracy}-\text{complexity}}{E_{q}\left[\log p\left(y|\theta ,m\right)\right]-D_{\mathrm{KL}}\left[q\left(\theta \right)\|p\left(\theta |m\right)\right]}\leq \log p\left(y|m\right) \end{array}$$

Because the divergence terms (Kullback–Leibler divergence, $D_{\mathrm{KL}}$) can never be less than zero, the free energy is a lower bound on log evidence. The second equality shows that the free energy balances accuracy—that is, expected log likelihood under the approximate posterior $E_{q}\left[\log p\left(y|\theta ,m\right)\right]$—and complexity—that is, the divergence between the approximate posterior and prior, $D_{\mathrm{KL}}\left[q\left(\theta \right)\|p\left(\theta |m\right)\right]$. This means that maximising model evidence (a.k.a., marginal likelihood) automatically penalises overly expressive models with redundant free parameters that do not significantly improve model fit. The fitted models contain means, variances and covariances of model parameters (multivariate normal probability density over parameter posteriors). DCM is available as part of the open source statistical parametric mapping (SPM12) academic software, in which several neural mass model types, and inference and analyses methods are implemented (Friston et al. 2021).

The CMC is an implementation of a canonical microcircuit in the DCM context, which allows for evaluation of models (hypotheses) related to electrophysiological responses (Bastos et al. 2012; Pinotsis et al. 2013). It models laminar‐specific connectivity (Haeusler and Maass 2007) and differences in spectral characteristics between superficial and deep pyramidal cells (Bastos et al. 2012; Bosman et al. 2012; Buffalo et al. 2011; Haeusler and Maass 2007). As such, it is suited to model oscillatory dynamics (spectral densities) observable in vivo (Fedele et al. 2023; Pinotsis et al. 2017; Rosch et al. 2018). Concretely, the CMC consists of four coupled populations: superficial pyramidal, spiny stellate, inhibitory interneuron and deep pyramidal parameterised by population‐specific excitatory, inhibitory, and modulatory coupling strengths and time constants. The parameters—their priors and mechanistic roles—are biophysically informed (Bastos et al. 2012; Daunizeau et al. 2011), and represent summated effects of inter‐ (excitatory, inhibitory) and intralaminar (modulatory) connections. Each might be modulated by various individual neurotransmitters and therefore, there is no direct or unique mapping between specific parameters and neurotransmitter systems. To note, recurrent inhibitory self‐modulation connections were found to be less sensitive to perturbations, effectively controlling oscillatory dynamics, which results in improved model evidence (Youssofzadeh et al. 2015). Individual population dynamics can be described by the following differential equation (neural model):

(3) $${\dot{v}}_{n}=i_{n}$$

(4) $$\begin{array}{l} {\dot{i}}_{n}=\underset{\text{tuned synaptic inputs}-\text{damping}-\text{decay}}{\frac{H_{n}}{\tau_{n}}\left(\sum d_{\mathrm{mn}}\sigma \left(v_{m}\right)\right)-\frac{2}{\tau_{n}}i_{n}-\frac{1}{\tau_{n}^{2}}v_{n}} \end{array}$$

Here $v_{n}$, $i_{n}$ and $\tau_{n}$ constitute membrane potential, current and time constant for a subpopulation *n*, and ${\dot{v}}_{n}$, ${\dot{i}}_{n}$ denote their time derivatives respectively. The synaptic response of a population is a function of adjusted summed presynaptic inputs $d_{\mathrm{mn}}\;\sigma \left(v_{m}\right)$ decreased by kinetics‐dependent damping $\left(2/\tau_{n}\right)i_{n}$ and decay $\left(1/{\tau^{2}}_{n}\right)v_{n}$. A single input in turn is the connectivity weighted $d_{\mathrm{mn}}$, sigmoid transformed postsynaptic depolarisation $\sigma \left(v_{m}\right)$ of a population *m* (Bastos et al. 2012; Moran et al. 2013; Pinotsis et al. 2013). Generally, this neural model describes electrical activity (membrane currents and potentials) on the cortical surface, and a separate observation model is applied, for example, a leadfield model that projects the activity of sources to scalp EEG sensors or local field potentials for iEEG. Steady state iEEG activity is modelled by fitting parameters to power spectral (or cross‐spectral) density summaries of oscillations. Effectively, this uses a local linear perturbation analysis around the fixed point of a nonlinear model (Moran et al. 2009).

For each of the 1770 iEEG recordings, we fitted an individual CMC in the frequency domain to the observed PSD. This delivered full posterior estimates in the neuronal population parameter space (three excitatory and three inhibitory between population coupling strengths, four inhibitory/self‐modulatory strengths, and four population time constants), and a free energy estimate for each CMC. These fully fitted models were the basis for subsequent hierarchical modelling steps. Note, this assumes that the iEEG data can be regarded as being generated by a single canonical (i.e., normative) subject.

### 2.6 Parametric Empirical Bayes

Parametric Empirical Bayes (PEB) is a hierarchical Bayesian modelling approach (Friston et al. 2016, 2015; Zeidman et al. 2019) used for second (group or between DCM) level analyses and parameter estimation based on first level DCM parameter posteriors. The PEB general linear model (GLM) allows to incorporate regressors (covariates) to test hypotheses, explain between subject—here measurement or channel—variability and adjust first level parameters. Using a general formulation of a DCM neural model (5) for illustration, changes in latent states can be estimated using current states $x_{t}$, input $u_{t}$ and a set of first level parameters $\theta_{i}^{\left(1\right)}$. To subsequently explain variation between subjects (differences in first level parameter $\theta^{\left(1\right)}$), known $\theta^{\left(1\right)}$ are equated to a GLM with design matrix X (covariates) and to be estimated group‐level parameters $\theta^{\left(2\right)}$ (6). The design matrix X is composed of between‐subject $X_{B}$ and within‐subject effects $X_{W}$. Subsequent estimation of the PEB GLM delivers group‐level model evidence for a given hypothesis and allows to update first level parameters.

(5) $$\frac{dx_{t}}{\mathrm{dt}}=f\left(x_{t},u_{t},\theta_{i}^{\left(1\right)}\right)$$

(6)

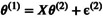




Here, PEB was employed to test hypotheses via changes in model evidence (free energy at the second level) after including neuroreceptor densities as explanatory variables (i.e., regressors), and to explore relations between neuroreceptor densities and first level CMC parameters. Additionally, hierarchical models tend to provide more robust estimates than first level parameters alone as they are constrained by group effects and are less likely to get stuck in local minima during model inversion.

To evaluate the effect of receptor density data on model evidence two PEB approaches were employed. The first PEB included densities of four candidate receptors (AMPA, NMDA, GABA_A_ and GABA_B_) as regressors to analyse the effect of each receptor type on model evidence (free energy). We evaluated all combinations of these RD regressors, that is, compared 15 different GLMs: four models with a single receptor density, six with two regressors, four with three regressors and one model with all four RD. We hypothesised that a mix of major excitatory and inhibitory RD would increase model evidence significantly.

For the second PEB, we first performed a PCA to capture cortical topographic variation and clusters of normalised RD in reduced dimension. This was helped by receptor fingerprint similarity of neighbouring, connected or functionally comparable areas (Zilles and Palomero‐Gallagher 2017) and by spatially co‐occurring receptor expression (Figure 3A,B, Figures S1, S2, and S8). Different features of the underlying receptor data are reflected in principal component (PC) gradients, for example, the distinctively low density of most receptors in the motor cortex is apparent in PC1 and higher expression of receptors for GABA_A_, NMDA, M_2_, alpha_2_, D_1_, 5‐HT_2_ in early visual areas in the occipital lobe is discernible in PC2 (Figure 3A, S8). The first one to six PC of the RD‐region matrix, which together explained 91% of variability (PC1 to 6 explained 0.405, 0.245, 0.106, 0.073, 0.048, 0.034, respectively) of the 15 RD across 44 regions, were used as regressors. This resulted in a 1770 × 7 matrix with group mean and PC covariates, and for this second PEB we combined the covariates in order—cumulatively, from the first to the sixth PC (PC1, PC1‐2, PC1‐3, PC1‐4, PC1‐5, PC1‐6). Thus, we inverted six second level models and used Bayesian model comparison for evaluation. This PEB was implemented as we reasoned that the entire population dynamics are only partially explained by the excitatory/inhibitory receptors in the first PEB and that variability in the composition of all regional RD—including neuromodulators—is informative and enhances fit.

To assess if improvements in model evidence are due to non‐specific spatial coherence among parameters, we used three independent sets of null models, which included the first four PCs to validate results of the overall winning model. For the first null model set (*N1*—receptor correlation preserved), values of all receptors together were randomly exchanged between regions which preserved the correlation of densities within a region. In the second null model variant (*N2*—regional boundary preserved), receptor values were exchanged between regions for each regressor individually, thus, it was only ensured that iEEG channels within a region have the same values for a given regressor. The last null models (*N3*—random) were unconstrained within a regressor, therefore, values of a receptor density were randomly shuffled between iEEG channels, which implies that channels in the same region likely have different receptor values. With the first null model set, the association between regional densities and observed dynamics was tested. With the second, the contribution of regional composition, that is, correlations between densities, was evaluated. And with the third, additionally, the importance of within‐region consistency was assessed. For each of the null model variants, regressor values were shuffled 40 times, delivering a representative number of individual model inversions per set for comparison.

Further, to contrast receptor density and CMC posterior profiles of periodic and aperiodic iEEG, we selected samples of 10 periodic iEEG recordings with the greatest spectral power for each canonical frequency band and 10 aperiodic iEEG spectra, which were defined as having the smallest linear mean squared error to the fractional noise (1/*f*) over the entire fitted range (1–60 Hz). The fractional noise was defined as a single line (intercept: 1, slope: −1 in logarithmic space), and was subtracted from all iEEG PSDs to obtain denoised traces. The traces were subsequently ordered by their remaining spectral power to illustrate commonalities in periodic iEEG oscillations and RD (Figures S1 and S2). To note, the generic 1/f line underestimates the noise in the delta band and assigns comparatively less weight to higher frequencies with lesser spectral power, resulting in less apparent (diffuse) trend correlations of the power in gamma frequencies and RD (Figure S2A,B).

## 3 Results

### 3.1 Neuronal Population Models Explain Regional Oscillatory Brain Dynamics

To describe the genesis of regional cortical population dynamics, we fitted single neural mass models (CMC) using DCM (Bastos et al. 2012; Moran et al. 2013; Pinotsis et al. 2013) for each of the 1770 normative iEEG recordings (Figure 2A). The iEEG atlas included stereotactic EEG (SEEG) and electrocorticographic (ECoG) recordings of putatively normal brain regions in 106 individual epilepsy patients (Frauscher et al. 2018b). These model inversions (without receptor density priors) constituted the first level of the hierarchical approach; it served to estimate initial parameter posteriors and to evaluate model fits across the range of regional brain dynamics.

The source‐specific, four population neural mass models (Figure 2F) generated PSD (1–60 Hz, 1 Hz steps) with very good fit for most iEEG recordings with few exceptions: 42/1770 (2.4%) electrode contacts showed a noticeable error (MSE > 0.05) in frequency fit (MSE median = 1.04*10^−6^ and mode = 1.29*10^−11^) (Figure 2G). In addition, the modelled PSDs match previously reported profiles (Frauscher et al. 2018b) and showed characteristic lobe‐specific periodic features such as an alpha peak in the occipital lobe, but also indicated high intra‐region and inter‐subject variability (Figures 2D,E and S5).

### 3.2 Variation in Neuroreceptor Densities Explains Regional Differences in Neuronal Population Dynamics

Next, we tested whether furnishing the regional neural mass models (CMC) with information about neurotransmitter RD (Zilles and Palomero‐Gallagher 2017) improves their fits, as scored with variational (free energy) bounds on log model evidence (Friston et al. 2007; Neal and Hinton 1998). Since there is no one‐to‐one mapping between autoradiographic receptor density values and the synaptic parameters of the CMC, this relationship was inferred using a hierarchical PEB approach (Friston et al. 2016, 2015; Zeidman et al. 2019): this hierarchical model comprised the nonlinear DCM at the first level and a GLM over DCM parameters at the second (group) level. Here, the GLM regressors modelled variation in RD and the second level inversion estimated the effect of these regressors on first level (within region) parameters. Subsequently, models including different subsets or linear combinations of receptor data were compared to identify the most likely hierarchical model.

First, models that included only receptor density combinations of major excitatory (glutamatergic; AMPA and NMDA) and inhibitory (GABAergic; GABA_A_ and GABA_B_) receptors (Figure 3A) were considered, as these receptor types correspond most directly to the synaptic parameters of the DCM neural mass model. Additionally, an evident association of RD to iEEG spectral power (Figures 3C and S2A,B) and their previously reported dominant influence on MEG power bands (Hansen et al. 2022) motivated this selection. We separately fitted the hierarchical models including regressor combinations of one to four receptor types and compared their evidence using the free energy approximation. The winning model contained AMPA, NMDA and GABA_A_ RD (*ANGa*) with a group‐level free energy difference to the next best model of > 5, which corresponds to a posterior probability > 0.99 for *ANGa* over other models. The next best model was the full model ANGaGb, which includes four RD as regressors. Although this model might be more accurate, variational free energy penalises for complexity, that is, redundant parameters, which precludes overfitting. Therefore, since increase in accuracy does not offset the penalty from additional free parameters, it has lower model evidence.

In addition to determining a winning model, the stepwise testing of receptor combinations allows evaluating their contribution to explaining electrophysiological variability. It is noticeable that, for example, receptors which show spatial similarity, such as AMPA and GABA_B_, or NMDA and GABA_A_ receptors, cause similar improvements in model evidence, and combining these has little additional benefit. In contrast, combinations of receptors with distinct spatial profiles—such as AMPA and NMDA receptors—lead to significant “information gain”, in other words, model evidence increases as a result of improved accuracy afforded by non‐redundant regressors.

**FIGURE 4:**
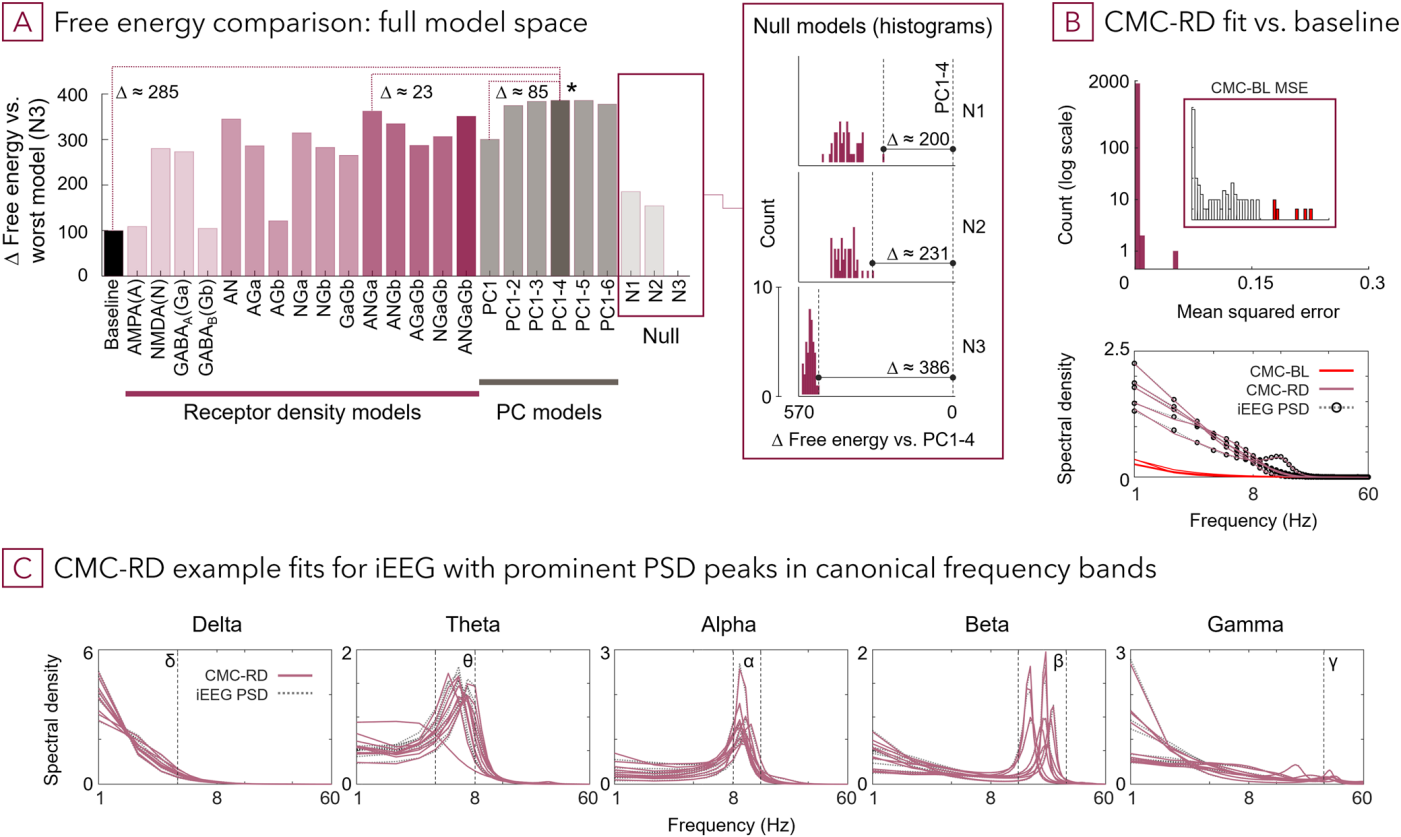
Prior information on the spatial distribution of neurotransmitter receptors improves generative models of intracranial cortical dynamics. (A) Comparison between baseline (standard CMC), candidate models and null models. Candidate models differ in which aspects of receptor density (RD) maps inform their inversion: Single main excitatory and inhibitory receptor types, combinations of main excitatory and inhibitory receptor types (A—AMPA, N—NMDA, Ga—GABA_A_, Gb—GABA_B_), for example, ANGa—PEB model with AMPA, NMDA, GABA_A_ receptor densities as regressors, or combination of n PC across all receptor types, included principle components are cumulative, for example, PC1‐6 is a PEB with 1 to 6 PCs as regressors. Null models (N1, N2, N3) have differently shuffled (randomised) four PCs as regressors; Inset: Histograms of PEB (group‐level) free energy values obtained from single runs for each null model set (N1—receptor correlation preserved, N2—region boundary preserved, N3—random) using randomised regressor values of the winning model (PC1‐4); deltas indicate the difference between the winning model and the best null model in each set after 40 randomisations. (B) Re‐estimating first level CMCs with coupling parameter priors of the winning PC1‐4 model (CMC‐RD) results in better fitted PSDs (1/1770, MSE > 0.05) (inset with CMC baseline [CMC‐BL] MSEs, indicated are worst fits [red]); plot below shows improvements for the five worst CMC‐BL fits; lobar PSDs and additional examples of improved fits are shown in Figures S4–S6. (C) Examples for achieved fits using PC1‐4 model priors: For each band the 10 iEEGs with the greatest spectral power (canonical frequency band peak) are shown. Details relating to differences in RD, PC and coupling parameters of these periodic iEEG compared to aperiodic iEEG are available in Figure S3.

Second, we assessed whether low dimensional representations across all 15 RD improve fit compared to the excitatory/inhibitory (*E*/*I*) systems' models. Principal component analysis was applied to identify axes of maximal regional variance across the 15 RD (Figure S8); the correlation between RD motivated the PCA (Figures 3B and S2). Then, one to six of the resultant PCs were included in this second hierarchical PEB model. The winning model (CMC‐RD *PC1‐4 (short CMC‐RD)*, including components one to four) had a free energy difference of > 5 (posterior probability > 0.99) compared to the previous winning model *ANGa* (Figure 4A). In fact, all models with more than one PC outperformed the excitatory/inhibitory neuroreceptor models. This suggests that the PCs contained additional information—from the entire regional receptor density composition including neuromodulators—which is relevant to explain neuronal dynamics, but which was not included in the densities of predominant E/I neurotransmitter systems.

The CMC with PC1‐4 (CMC‐RD) captured spectral power peaks in canonical frequency bands more accurately and successfully avoided local optima which partially caused fitting errors in the baseline model (Figure 4B, detailed in: S4 CMC‐BL vs. CMC‐RD, S5 lobar fits, S6 best/worst fits). Sampled periodic iEEG with significant power peaks showed distinct profiles for RD and for model parameters compared to aperiodic iEEG on average (Figure S3). Across canonical bands, neuroreceptor expressions were predominantly higher in oscillatory iEEG. Spatial overlaps to regionally distinct brain rhythms were established (Capilla et al. 2022; Hansen et al. 2022; Siebenhühner et al. 2024), and influences of upregulated receptors can be observed, for example, AMPA‐R for delta and gamma frequencies, or GABA_A_‐R for theta and alpha rhythms. For CMC‐RD parameters, visual inspection suggests that oscillatory (PSD) similarity is reflected in average posterior profiles, even across frequency band boundaries (Figure S3). For example, several CMC coupling parameters for iEEG with theta or alpha peaks show similar deviations to aperiodic iEEG models.

Lastly, we evaluated whether the effects of receptor density information on model evidence could be explained away by spatial factors rather than by the distributions of receptor data. Since the receptor density estimates are spatially smooth and neighbouring cortical areas are likely to show more similar iEEG signals than those further apart, the addition of random but spatially smooth priors may improve model evidence. This was tested by inverting three null model sets through constrained shuffling of the receptor density values for each brain region: *N1*, shuffling while preserving correlation between regressors (RD) within individual brain regions; *N2*, shuffling while preserving brain region boundaries; and *N3*, randomly shuffling of all receptor density values amongst iEEG channels individually. As expected, some of these null models outperformed an uninformed baseline model, but all had a significantly lower model evidence than the original winning models (ANGa and PC1‐4) and any of the PCA models (Figure 4A).

## 4 Discussion

In this study, we demonstrated that neurotransmitter receptor densities shape resting state intracranial EEG dynamics. Normative receptor data and regional iEEG spectra were related to the parameters of a biophysically inspired generative model of cortical oscillations through an iterative Bayesian procedure. This resulted in an atlas of neurobiologically informed synaptic parameters (i.e., intrinsic connectivity) for neuronal populations. These results speak to a central neuroscience question: how observable brain signals such as intracranial EEG power spectra can be explained by attributes of—and variations in—underlying neurobiology or structure (Avena‐Koenigsberger et al. 2017; Kopell et al. 2014; Shine et al. 2021; Suárez et al. 2020). Specifically, we provided quantitative evidence that neurotransmitter systems and their interactions underwrite emergent neuronal dynamics and that their influence can be captured with generative (neural mass) models. Additionally, we illustrated an efficient approach to translate across spatial scales in measures of brain structure and function in health and disorders.

### 4.1 Receptor Densities Shape Regional Electrophysiology

We focussed on assessing the explanatory power of cortical neurotransmitter receptors as key mediators of signal transduction between neurons (Bennett 2000; Lovinger 2008; Shine 2019). Aggregated regional receptor compositions represent a useful descriptor with molecular specificity, which may approximate meso‐ and macroscale electrophysiology (Goulas et al. 2021; Zachlod et al. 2023; Zilles and Palomero‐Gallagher 2017); and topographic overlap of receptor density data and electrophysiological frequency bands was previously shown statistically (Hansen et al. 2022). The generative modelling approach presented here, offers mechanistic evidence that regional variability in receptor composition can explain differences in neuronal dynamics, when mediated through nonlinear models of population dynamics. It enabled us to find a parametric mapping of how spectral variation in low noise iEEG relates to the underlying regional composition of neurotransmitter receptor density and to obtain normative models across the adult human brain.

### 4.2 Mesoscale Models Mediate Between Microscale Synaptic Properties and Emergent Macroscale Brain Dynamics

Regional receptor density compositions and iEEG signals are situated at millimetre scale patches of cortical layers. The abstraction from individual microscopic structural components, such as neuronal cells, transmitters or receptors, allows to focus on emergent dynamics at population and circuit level which subsume underlying interactions such as modulatory or compensatory up‐ and downregulation of specific neuronal receptor subtypes (Palomero‐Gallagher et al. 2012). This means that quantitative representations of cortical dynamics such as PSD can be aptly modelled using coupled neuronal populations: for instance, the DCM canonical microcircuit neural mass model. Furthermore, Bayesian inversion facilitates data‐driven learning of the interrelation between RD and CMC parameters. Employing this technique, we found that including entire receptor density compositions via PCs significantly improved models compared to incorporating only predominant excitatory and inhibitory neuroreceptors.

Further, changes in PSD obtained from iEEG can be linked to CMC parameter variations, and PEB coefficients (Figure S8B) combined with PCA gradients allow to assess the influence of PCs onto regionally specific CMC parameters. But since PCs capture the shape of variation in normalised RD and since the effects of the CMC parameter changes are accumulative, interpreting PSDs in terms of contributions of individual neurotransmitter receptors requires further testing. Overall, our results suggest that neuronal dynamics emerge from interacting neurotransmitter systems and are only partially determined by the excitatory‐inhibitory balance (Luppi et al. 2023). In essence, mesoscale models with limited but sufficiently complex parameter sets seemingly offer an appropriate trade‐off between complexity and tractability for modelling regional and whole‐brain dynamics.

### 4.3 Towards a Normative Atlas of Synaptic Population Parameters of Cortical Dynamics

In addition to demonstrating mechanistic spatial dependencies between RD and cortical dynamics, we provide a normative set of generative neural mass models across the cortical surface. Incorporating connectivity and biophysical properties of populations as well as additional regional receptor information, the ensuing synaptic parameter estimates represent a theoretically and practically relevant annotation of the human cortex, which may inform future DCM studies. Within the DCM framework, hidden neuronal sources—modelled as interacting populations—may be equipped with parameter priors and then fitted to EEG and MEG datasets in both time and frequency domains. Contextualised broadly, our approach—and the availability of the cortical parameter atlas—extends decade long modelling of brain dynamics with neural mass models (Destexhe and Sejnowski 2009; Glomb et al. 2022).

Incorporating the regional chemoreceptor composition into neural mass models to explain measured electrophysiology provides an opportunity to understand brain dynamics in terms of bottom‐up and top‐down causation. We envision that this allows to establish further links between chemoarchitecture and (clinically) measurable electrophysiology both in health and disorders. Consequentially, it may facilitate, for example, modelling and understanding of the entangled effects pharmacological compounds exert on different neurotransmitter systems. Our neurobiologically informed CMC seems suited to study these complex interactions and their electrophysiological signatures, as it accounts for the interrelation of 15 neuroreceptors, while generating larger‐scale signals. Therefore, questions of how a drug affects (human) brains regionally or entirely through modulation of the underlying transmitter systems can be studied and explained. Hence, this approach complements biologically detailed computational models in drug discovery research as it abstracts from molecular and cellular mechanisms (Chang et al. 2022; Lin et al. 2020) and instead models the consolidated effects compounds exercise onto the brain regionally or globally. In other words, it models characteristic emergent phenomena (Hoel et al. 2013) taking various spatial scales and details into account.

### 4.4 Limitations and Constraints

The computationally efficient implementation within DCM allows evaluating various hypotheses or explanations effectively. However, this comes at the expense of the variational Bayes approach being susceptible to locally—but not globally—optimal solutions (Aponte et al. 2022; Cooray et al. 2023). In addition, since parameters collapse various underlying neuronal mechanisms, one‐to‐one reverse mapping requires elaborate testing procedures and might only provide approximate answers, potentially limiting predictive validity, for example, for explaining the effects of pharmacological interventions without further data. Thus, the conclusions and inferences rest upon the candidate models considered, or in other words, there is no intimation that the model selected is necessarily the best model; it just provides the most appropriate explanation among the models or hypotheses entertained.

Another limitation of this study relates to the normative data used, as both the iEEG and the autoradiography datasets constitute only relatively small and spatially (regional and laminar‐specific) averaged samples from which inferences on normative electrophysiology and chemoarchitecture were drawn. In consequence, the presented modelling approach reflects—and is affected by—the resolution of available normative datasets. Additionally, the choice of the mesoscale neural mass model might influence fit and parameter posteriors. Despite these particularities, we expect that the main result of the study is robust against modelling choices; namely, evidence for an association between the spatial distribution of iEEG features and RD at macroscopic spatial scales. And we hope that the accessibility of the Bayesian framework used here will allow future studies to statistically evaluate alternative models against the conclusions presented in this work.

We chose the DCM canonical microcircuit as a well‐studied and commonly applied convolution‐based neural mass model. Its implementation in a standard software package helps to replicate and extend our approach. Future DCM studies implementing the CMC can therefore easily use the derived priors or might further refine our findings and integrate additional prior information (regressors) iteratively using the Bayesian belief‐updating procedures described above. To establish relationships between spatial receptor density profiles and electrophysiology, it would be particularly helpful to use more granular (mm‐scale and laminar‐specific) receptor density data. In addition, mechanistic explanations might benefit from including complementary EEG/MEG data to characterise whole‐brain network dynamics, and including PET data to study the effects of in vivo alterations related to specific receptors.

### 4.5 Empirically Informed Multimodal Models of Brain Dysfunction May Aid Personalised Medicine and Novel Therapeutic Approaches

We have demonstrated an approach that uses Bayesian inference to link multimodal spatial datasets through generative models of brain function. This flexible methodology has wide‐ranging applicability in neuroscience research; especially, since the capacity to evaluate brain (dys)function with complementary modalities at varying spatial scales increases (Amunts et al. 2016, 2013, 2020; Ding et al. 2016; Hawrylycz et al. 2012; Horien et al. 2021; Larivière et al. 2021; Markello et al. 2022). In the future, this might have practical implications as it could help to establish in silico models to predict changes in whole‐brain dynamics following therapies with molecular targets. Such models would aid in stratifying patients into aetiological groups according to electrophysiological phenotypes and support decision‐making in empirically informed, personalised interventions for brain dysfunction.

## 5 Data, Materials, and Software Availability

Functions and models can be found in the statistical software package for neural imaging and electrophysiological data ‘Statistical Parametric Mapping’, Version 12 (Friston et al. 2021) implemented in MathWorks MATLAB.

## Supporting information


**Figure S1:** iEEG spectral power and receptor densities. In all subplots the iEEG channel spectra are sorted in ascending order (left to right) of increasing power in the entire modelled frequency range of 1–60 Hz. The receptor densities at iEEG locations (regions) are *z*‐scored and averaged (simple moving average, window size = 100 channels, *x* ± 50) to achieve smoothing and to visualise trends (white lines).
**Figure S2:** iEEG canonical frequency band spectral power and receptor densities. (A) The top row shows iEEG PSDs sorted by power in the respective frequency band (ascending left to right), which is shown in more detail in the row below. (B) Averaged (moving average, window size = 200) *z*‐scores of the receptor densities, ordered in accordance with the iEEG channels in (A). (C) The first four principal components, *z*‐scored and averaged as the RD, and sorted as the iEEG in (A).
**Figure S3:** iEEG oscillations (peaks), variation in receptor densities, principal components and CMC coupling parameters (posteriors). (A) Ten iEEG with the greatest spectral power in the respective canonical frequency band (observations and fit of the CMC with PC1‐4 priors (CMC‐RD)). (B) Difference in receptor densities between peak (periodic) and aperiodic iEEG traces, calculated as (RD average of the 10 iEEG with the highest band power) minus (RD average of the 10 iEEG which have the smallest root mean squared error to 1/*f*). (C) Similar to (B), here the periodic‐aperiodic difference in means of the RD derived PCs is shown. (D) The difference in means between the DCM CMC‐RD coupling parameter posteriors of the 10 periodic iEEG and the 10 aperiodic iEEG is shown; the grey bar is a visual assistance indicating the range [−0.15; 0.15].
**Figure S4:** Fit comparison for oscillatory peaks in canonical frequency bands. (A) Individual panels show example fits for 20 iEEG power spectral densities: the top row panels show the fits of the CMCs with PC1‐4 priors (CMC‐RD), the middle panels show CMC baseline (CMC‐BL), the bottom row shows the median and mean (dotted lines) differences (∆) between CMC‐RD (red)/CMC‐BL (black) fitted spectra and observed iEEG PSD. (B) The same data illustrated in more detail, that is, the plots only show the frequency range of the canonical bands (scales adjusted).
**Figure S5:** Lobar iEEG spectral densities, baseline and receptor density informed model fits. Left panels: spectra (dark grey) and maximal frequency power (light grey) of all iEEG recordings show intra‐ and interregional variability. Some distinct regional peaks are visible, such as the alpha frequency (8–12 Hz) peak in occipital lobe regions. Middle and right panels show iEEG spectra estimates by the DCM CMC baseline models and CMCs with priors of the winning PEB model with four principal component regressors (PC1‐4) respectively (dark red lines), with maximal power observations (light red area). Examples of improved fits are indicated with blue arrows.
**Figure S6:** Examples for fit improvements achieved in the CMC with PC1‐4 priors. (A) Examples of good and bad CMC model fits: the 25 best (lowest MSE) and 5 worst fits (largest MSE) of the baseline DCM CMC (CMC‐BL) (B) are compared against the fits to the same iEEG traces after including the priors of the PC1‐4 model (CMC‐RD). Particular improvements of spectral fits in lower frequencies (see also Figure S5).
**Figure S7:** Parameter posterior comparison and lobar parameters. (A) Comparison of DCM CMC coupling parameter posterior (stored as Ep.G in a SPM DCM structure data type for a CMC) after fitting 1770 baseline models (left) and CMCs with neuroreceptor priors (PC1‐4) (right) respectively. Shown are the values/ranges of posteriors (red‐excitatory, blue‐inhibitory, black‐modulatory) across regions with overall unweighted averages (grey circles). (B) Lobar densities of coupling parameter posteriors for the 1770 CMCs and averages (black dots) with RD‐informed priors—coupling parameter priors of the winning PEB PC1‐4 model—by lobe (medial and lateral frontal regions are combined in frontal).
**Figure S8:** Regional receptor densities and PEB group level parameters. (A) Receptor densities and brain regions (Broadmann areas and lobes). Region labels were named in accordance with the autoradiography study from which the receptor density data were taken (Zilles and Palomero‐Gallagher 2017). (B) PEB (group‐level) estimates for neural population parameters covary with the receptor density principal components 1–4. The values indicate weightings of PEB regressors and cause relative adjustments of first level parameters versus group means in parameter space; right the underlying CMC model. Updated DCM (first level) posteriors are shown in Figure S3. Combining the mapped regional coefficients for each channel as part of the design matrix (Figure S4) and PEB group level parameters gives estimates for first level DCM parameters.

**Table S1:** Neurotransmitter receptors. List of receptors included in the autoradiography study (Zilles and Palomero‐Gallagher 2017).

## Data Availability

Code and data are available upon reasonable request. Following open source data were used: intracranial EEG (Frauscher et al. 2018a, 2018b) and cortical receptor densities (Zilles and Palomero‐Gallagher 2017). Our code is based on the open source, academic software SPM 12 (Friston et al. 2021) built for MATLAB/Octave.
