# Supplementary material for: Topographic Variation in Human Neurotransmitter Receptor Densities Explains Differences in Intracranial EEG Spectra
Source: Hum Brain Mapp. 2025 Oct 31;46(16):e70393. doi: 10.1002/hbm.70393 (PMC12576962; doi:10.1002/hbm.70393)
Supplement: Supplementary file 1 — Figure S1: iEEG spectral power and receptor densities. In all subplots the iEEG channel spectra are sorted in ascending order (left to right) of increasing power in the entire modelled frequency range of 1–60 Hz. The receptor densities at iEEG locations (regions) are z‐scored and averaged (simple moving average, window size = 100 channels, x ± 50) to achieve smoothing and to visualise trends (white lines). Figure S2: iEEG canonical frequency band spectral power and receptor densities. (A) The top row shows iEEG PSDs sorted by power in the respective frequency band (ascending left to right), which is shown in more detail in the row below. (B) Averaged (moving average, window size = 200) z‐scores of the receptor densities, ordered in accordance with the iEEG channels in (A). (C) The first four principal components, z‐scored and averaged as the RD, and sorted as the iEEG in (A). Figure S3: iEEG oscillations (peaks), variation in receptor densities, principal components and CMC coupling parameters (posteriors). (A) Ten iEEG with the greatest spectral power in the respective canonical frequency band (observations and fit of the CMC with PC1‐4 priors (CMC‐RD)). (B) Difference in receptor densities between peak (periodic) and aperiodic iEEG traces, calculated as (RD average of the 10 iEEG with the highest band power) minus (RD average of the 10 iEEG which have the smallest root mean squared error to 1/f). (C) Similar to (B), here the periodic‐aperiodic difference in means of the RD derived PCs is shown. (D) The difference in means between the DCM CMC‐RD coupling parameter posteriors of the 10 periodic iEEG and the 10 aperiodic iEEG is shown; the grey bar is a visual assistance indicating the range [−0.15; 0.15]. Figure S4: Fit comparison for oscillatory peaks in canonical frequency bands. (A) Individual panels show example fits for 20 iEEG power spectral densities: the top row panels show the fits of the CMCs with PC1‐4 priors (CMC‐RD), the middle panels show CMC baseline [file HBM-46-e70393-s001.docx]

| 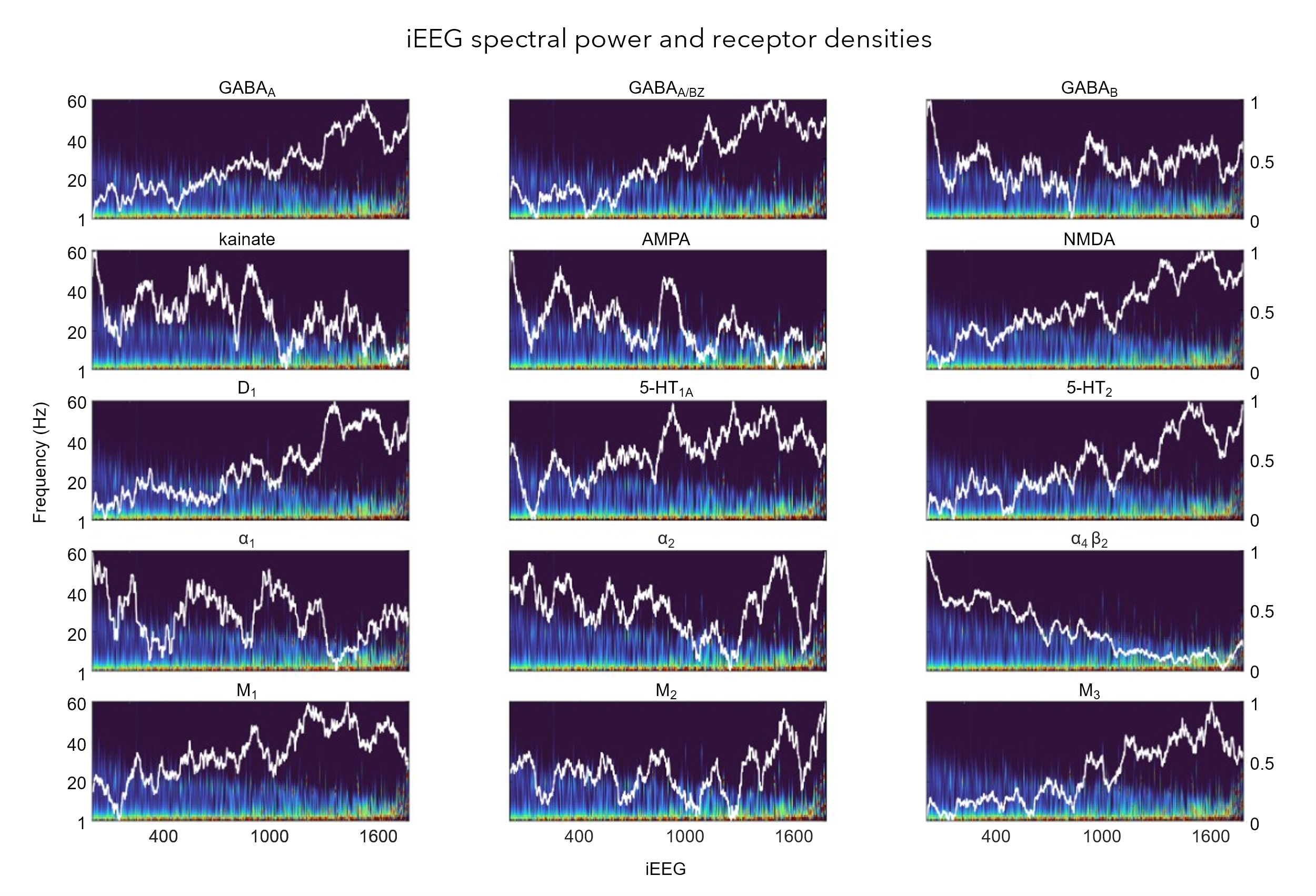 |
| --- |
| ***Supplementary Figure S1. iEEG spectral power and receptor densities.*** *In all subplots the iEEG channel spectra are sorted in ascending order (left to right) of increasing power in the entire modelled frequency range of 1-60Hz. The receptor densities at iEEG locations (regions) are z-scored and averaged (simple moving average, window size = 100 channels, x ± 50) to achieve smoothing and to visualise trends (white lines).* |

| 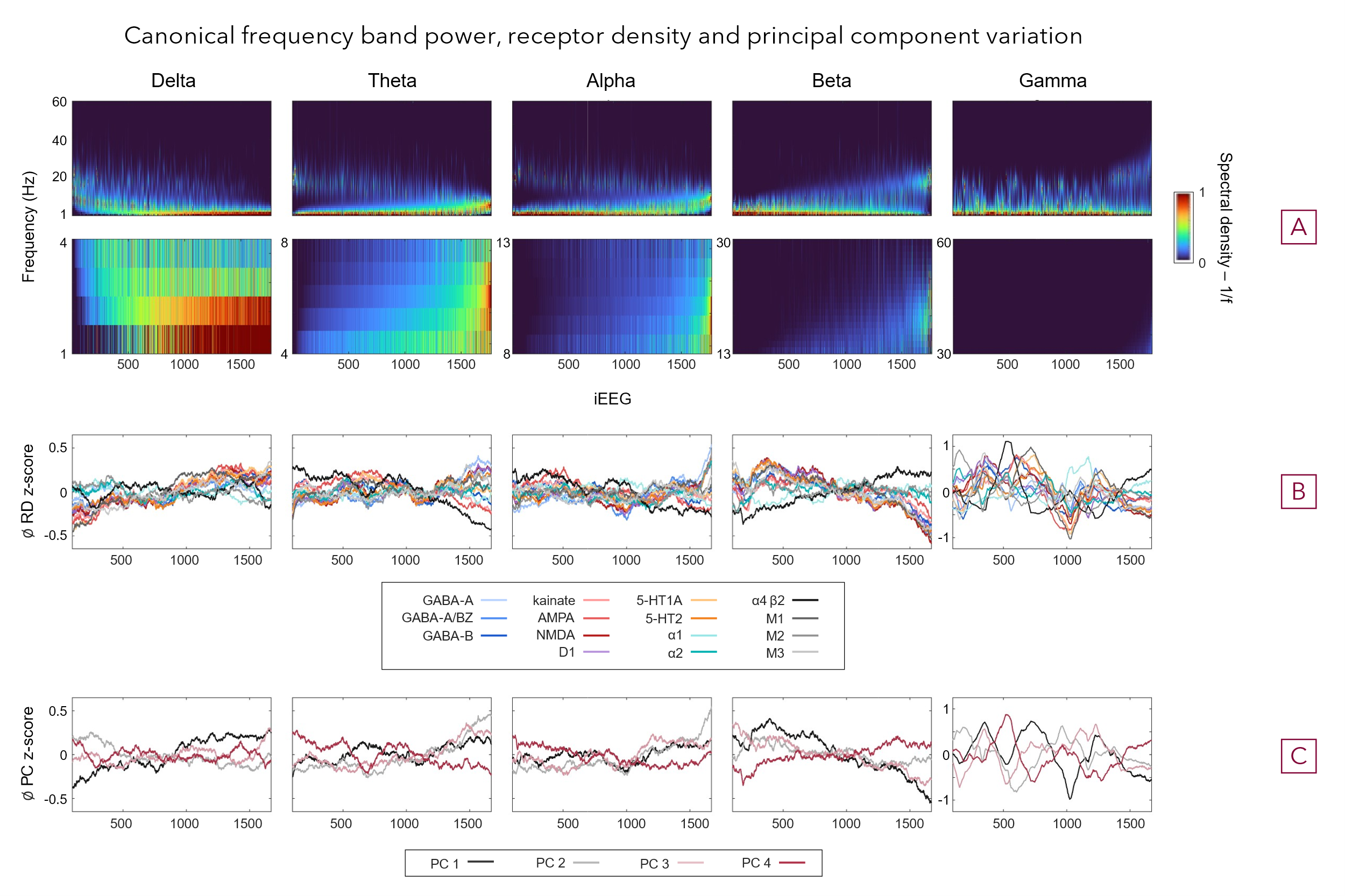 |
| --- |
| ***Supplementary Figure S2. iEEG canonical frequency band spectral power and receptor densities. (A)*** *The top row shows iEEG PSDs sorted by power in the respective frequency band (ascending left to right), which is shown in more detail in the row below.* ***(B)*** *Averaged (moving average, window size = 200) z-scores of the receptor densities, ordered in accordance with the iEEG channels in (A).* ***(C)*** *The first four principal components, z-scored and averaged as the RD, and sorted as the iEEG in (A).* |

| 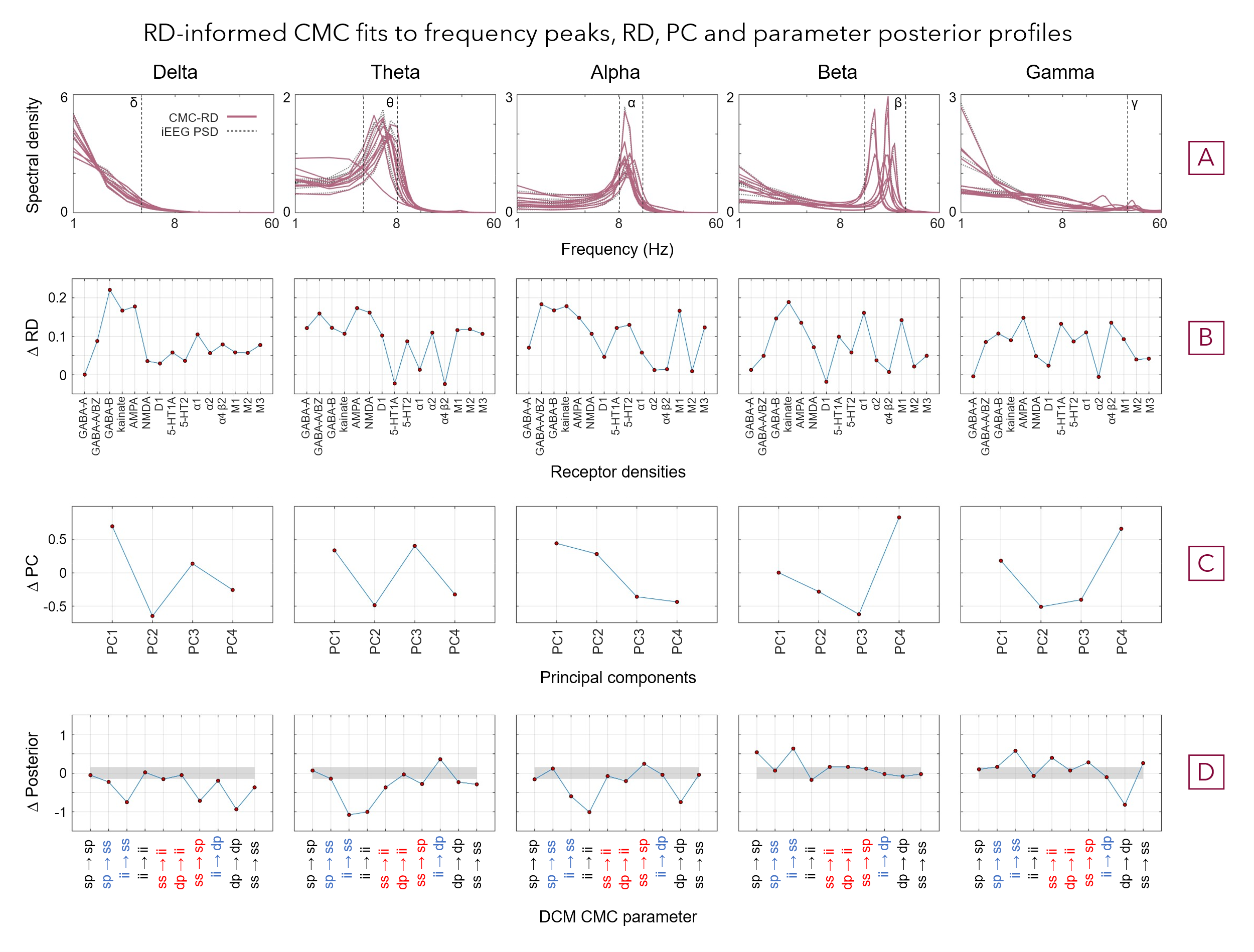 |
| --- |
| ***Supplementary Figure S3. iEEG oscillations (peaks), variation in receptor densities, principal components and CMC coupling parameters (posteriors). (A)*** *Ten iEEG with the greatest spectral power in the respective canonical frequency band (observations and fit of the CMC with PC1-4 priors (CMC-RD)).* ***(B)*** *Difference in receptor densities between peak (periodic) and aperiodic iEEG traces, calculated as (RD average of the 10 iEEG with the highest band power) minus (RD average of the 10 iEEG which have the smallest root mean squared error to 1/f).* ***(C)*** *Similar to (B), here the periodic-aperiodic difference in means of the RD derived PCs is shown.* ***(D)*** *The difference in means between the DCM CMC-RD coupling parameter posteriors of the 10 periodic iEEG and the 10 aperiodic iEEG is shown; the grey bar is a visual assistance indicating the range [-0.15; 0.15].* |

| 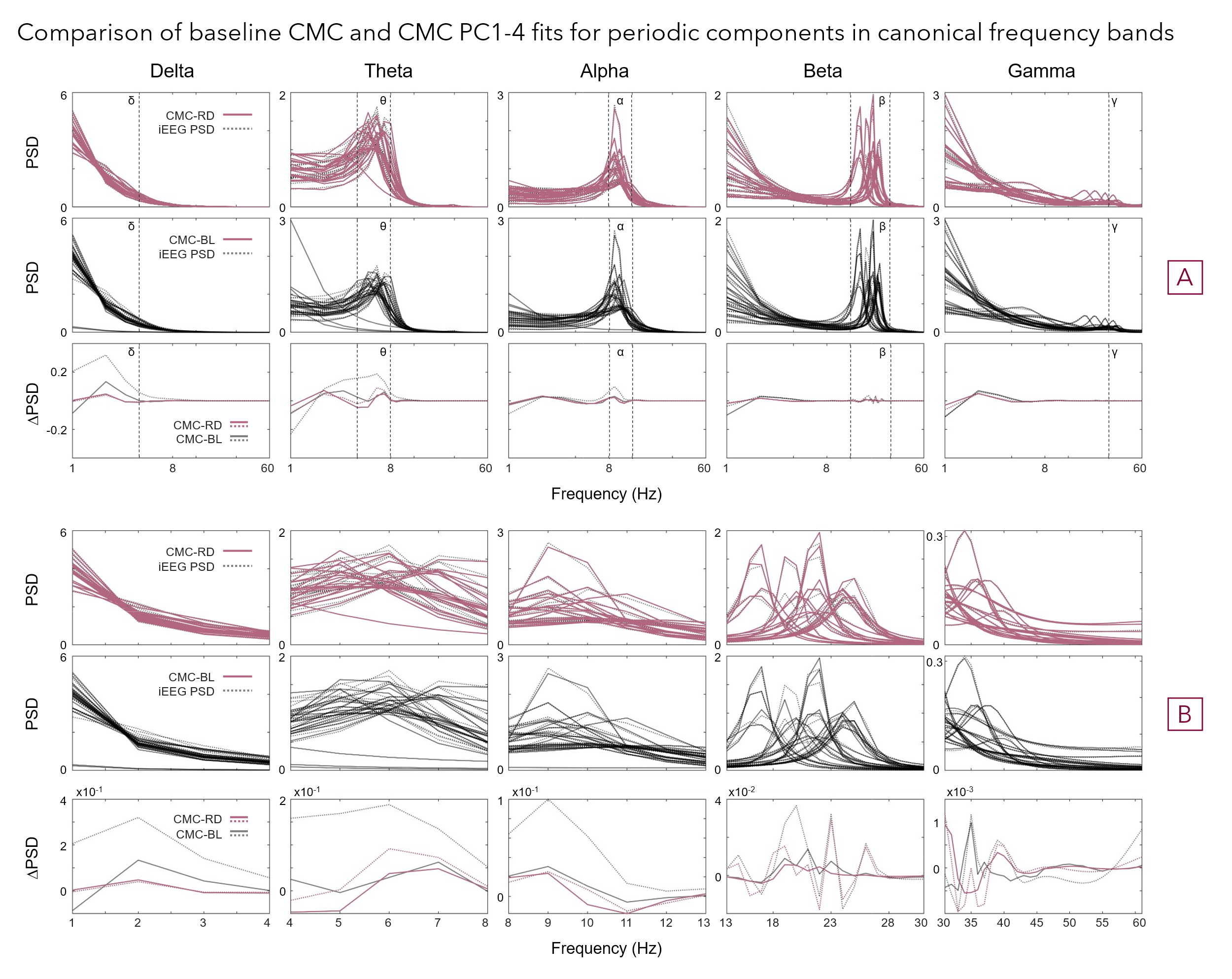 |
| --- |
| ***Supplementary Figure S4. Fit comparison for oscillatory peaks in canonical frequency bands. (A)*** *Individual panels show example fits for 20 iEEG power spectral densities: the top row panels show the fits of the CMCs with PC1-4 priors (CMC-RD), the middle panels show CMC baseline (CMC-BL), the bottom row shows the median and mean (dotted lines) differences (∆) between CMC-RD (red) / CMC-BL (black) fitted spectra and observed iEEG PSD.* ***(B)*** *The same data illustrated in more detail, i.e., the plots only show the frequency range of the canonical bands (scales adjusted).* |

| 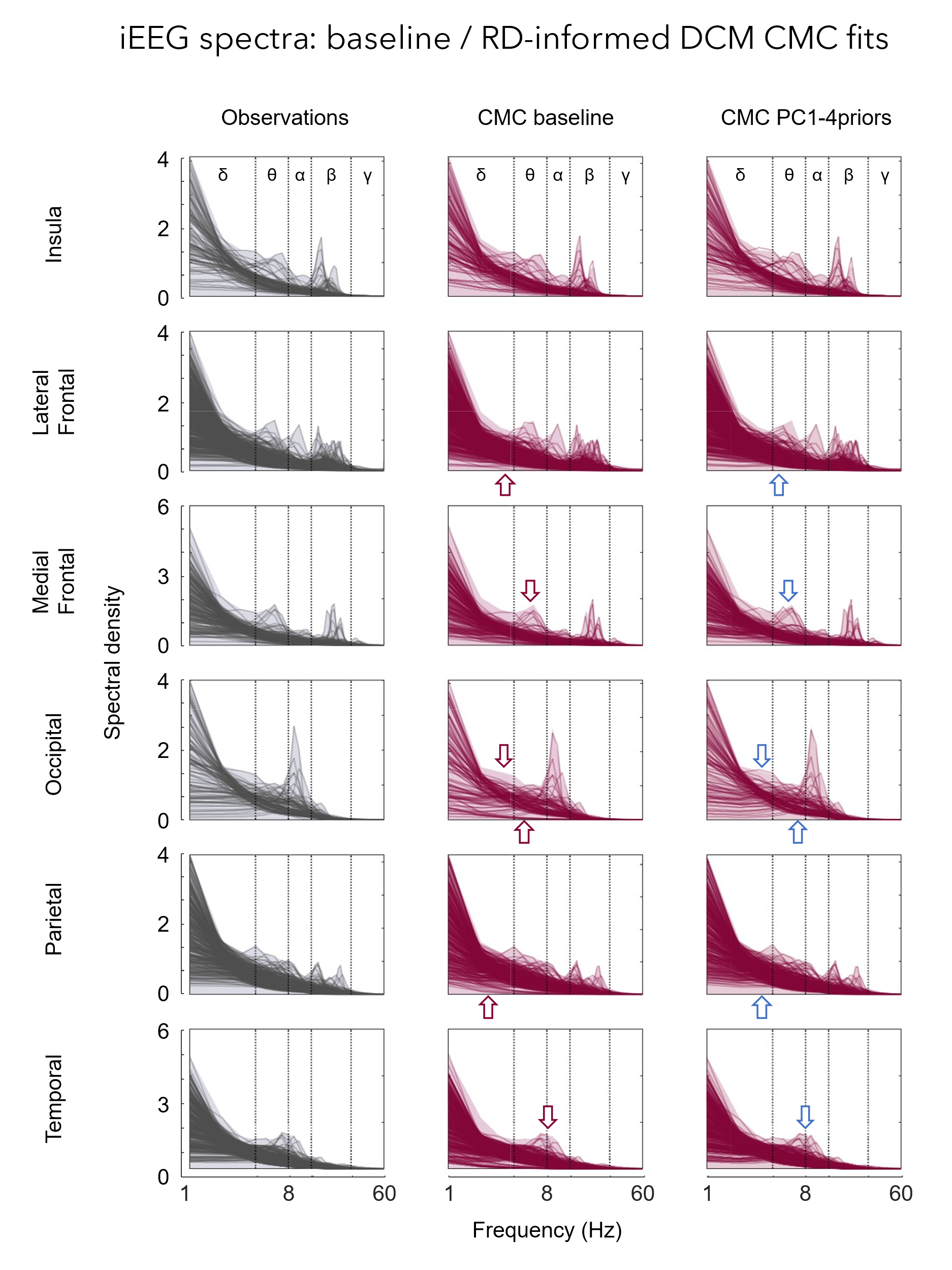 |
| --- |
| ***Supplementary Figure S5. Lobar iEEG spectral densities, baseline and receptor density informed model fits.*** *Left panels: spectra (dark grey) and maximal frequency power (light grey) of all iEEG recordings show intra- and interregional variability. Some distinct regional peaks are visible, such as the alpha frequency (8-12Hz) peak in occipital lobe regions. Middle and right panels show iEEG spectra estimates by the DCM CMC baseline models and CMCs with priors of the winning PEB model with four principal component regressors (PC1-4) respectively (dark red lines), with maximal power observations (light red area). Examples of improved fits are indicated with blue arrows.* |

| 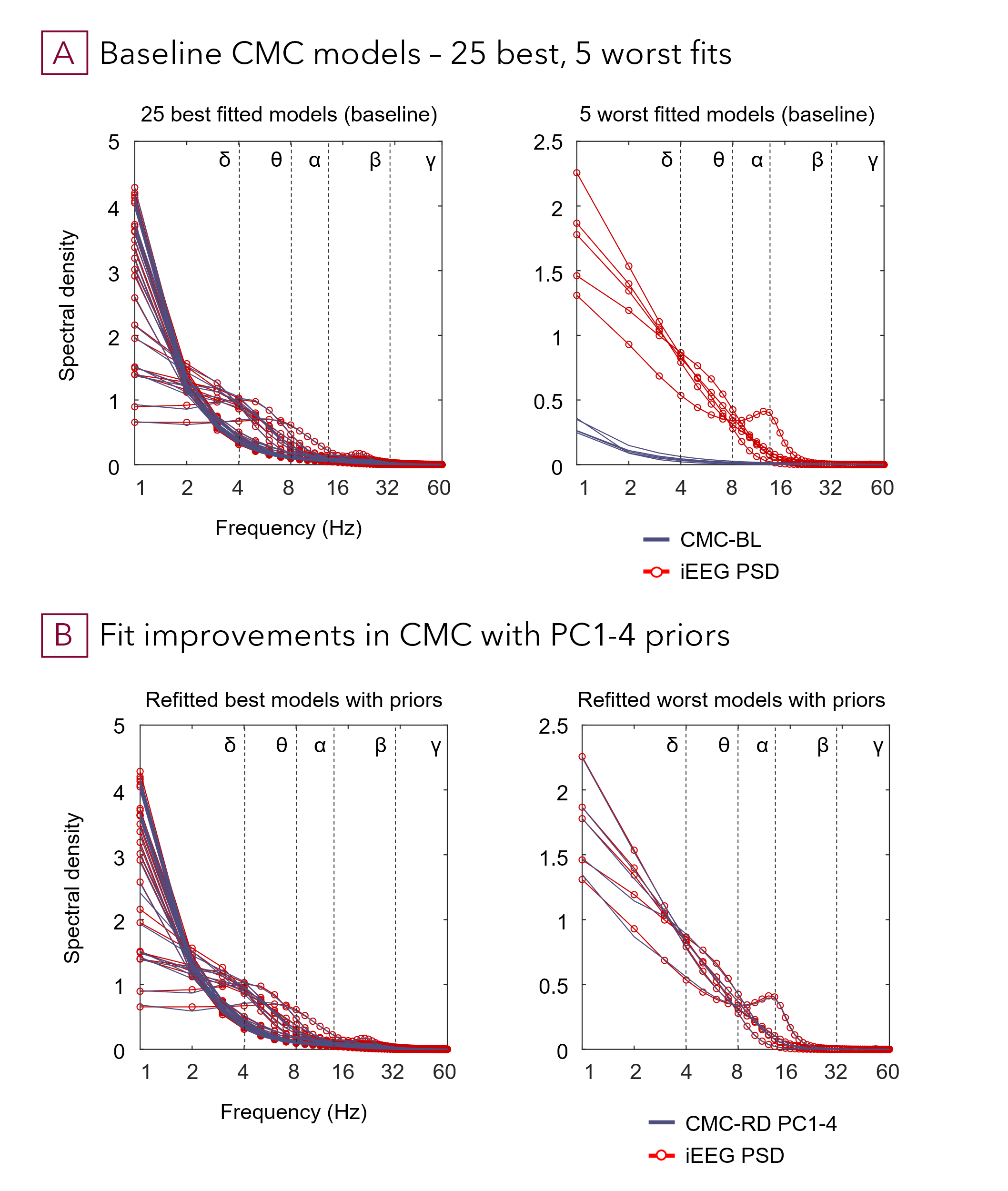 |
| --- |
| ***Supplementary Figure S6. Examples for fit improvements achieved in the CMC with PC1-4 priors. (A)*** *Examples of good and bad CMC model fits: the 25 best (lowest MSE) and 5 worst fits (largest MSE) of the baseline DCM CMC (CMC-BL)* ***(B)*** *are compared against the fits to the same iEEG traces after including the priors of the PC1-4 model (CMC-RD). Particular improvements of spectral fits in lower frequencies (see also Figure S5).* |

| 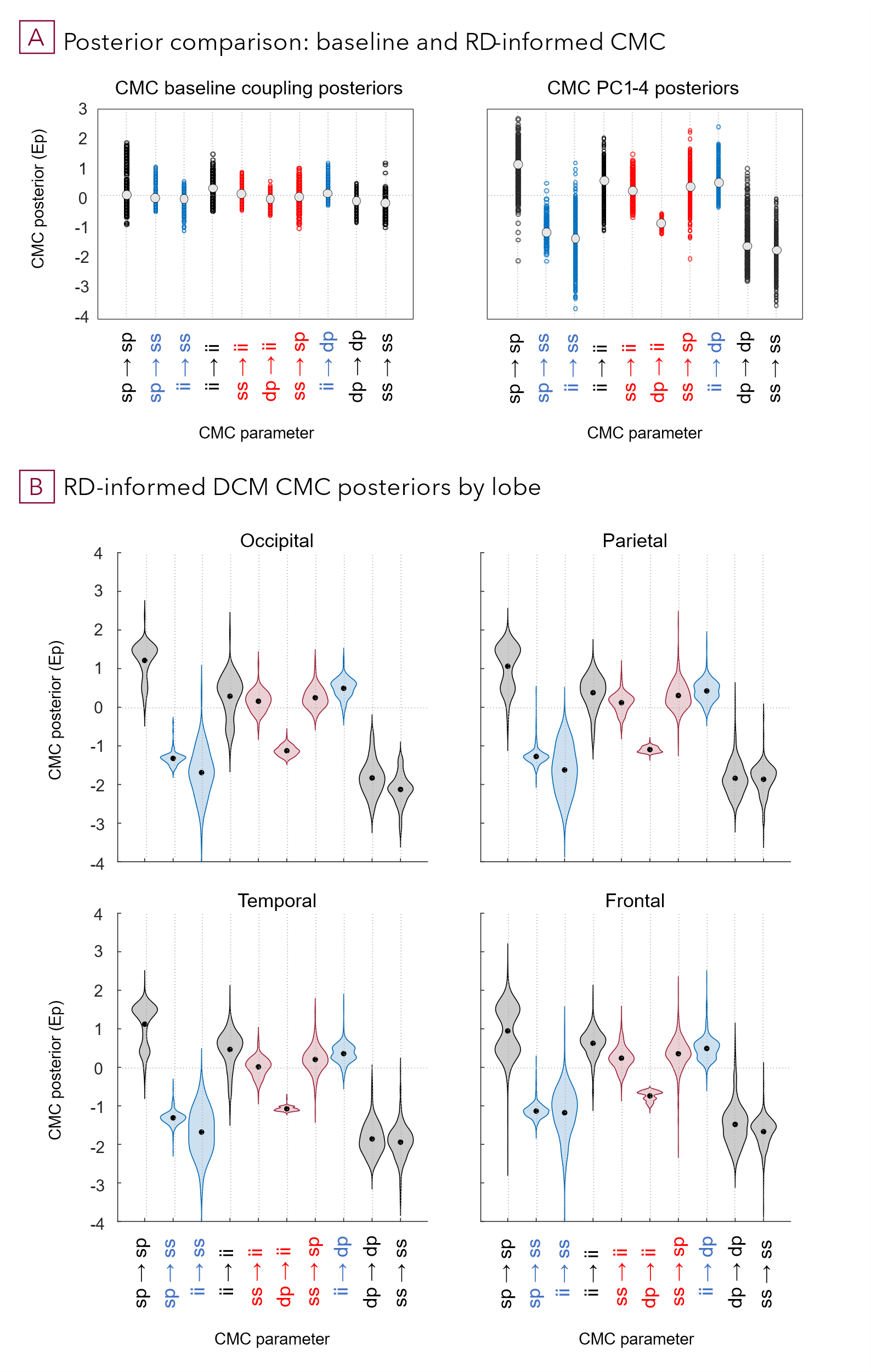 |
| --- |
| ***Supplementary Figure S7. Parameter posterior comparison and lobar parameters. (A)*** *Comparison of DCM CMC coupling parameter posterior (stored as Ep.G in a SPM DCM structure data type for a CMC) after fitting 1770 baseline models (left) and CMCs with neuroreceptor priors (PC1-4) (right) respectively. Shown are the values / ranges of posteriors (red-excitatory, blue-inhibitory, black-modulatory) across regions with overall unweighted averages (grey circles).* ***(B)*** *Lobar densities of coupling parameter posteriors for the 1770 CMCs and averages (black dots) with RD-informed priors – coupling parameter priors of the winning PEB PC1-4 model – by lobe (medial and lateral frontal regions are combined in frontal).* |

| 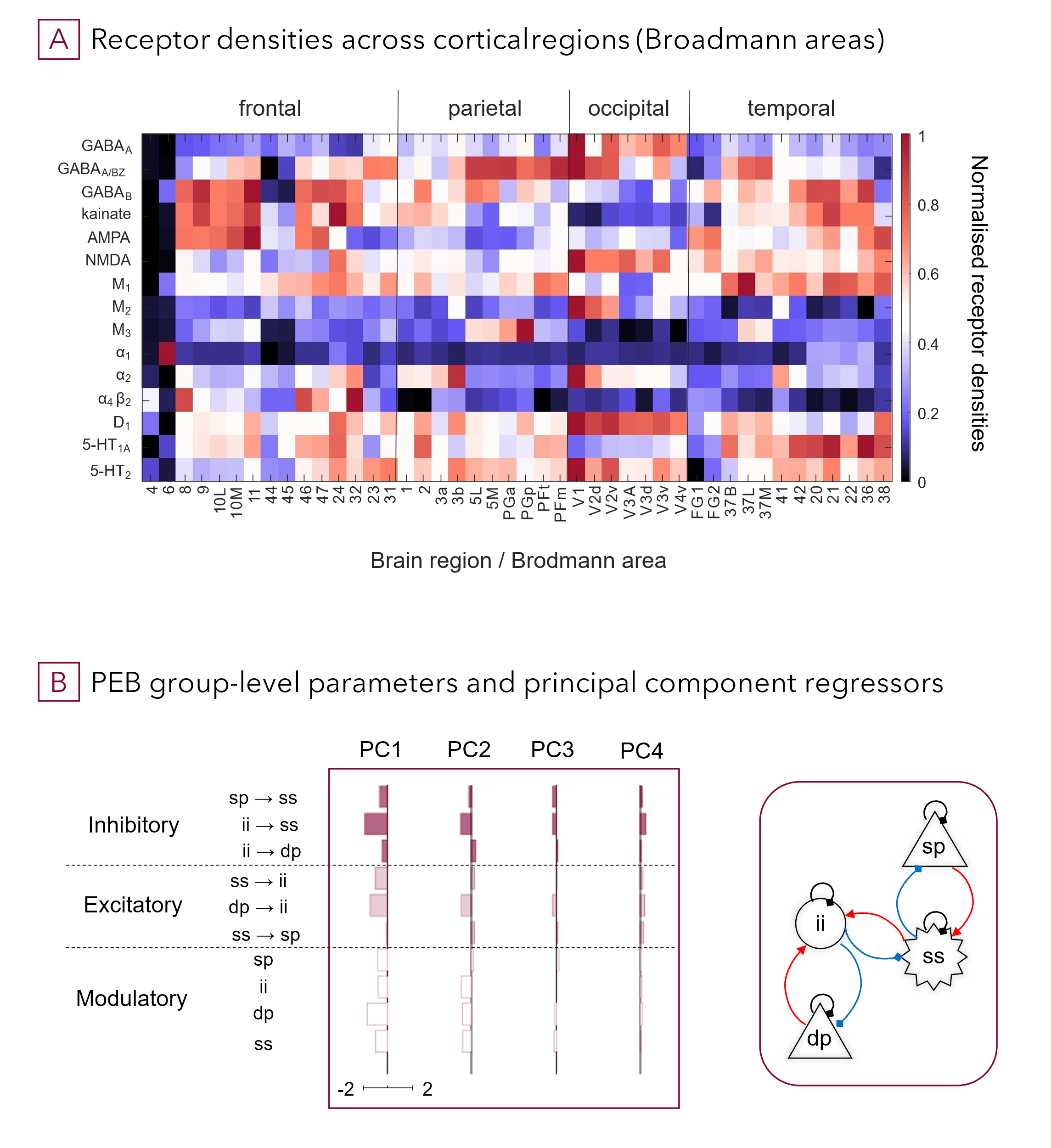 |
| --- |
| ***Supplementary Figure S8. Regional receptor densities and PEB group level parameters. (A)*** *Receptor densities and brain regions (Broadmann areas and lobes). Region labels were named in accordance with the autoradiography study from which the receptor density data were taken (Zilles & Palomero-Gallagher, 2017).* ***(B)*** *PEB (group-level) estimates for neural population parameters covary with the receptor density principal components 1-4. The values indicate weightings of PEB regressors and cause relative adjustments of first level parameters versus group means in parameter space; right the underlying CMC model. Updated DCM (first level) posteriors are shown in Figure S3. Combining the mapped regional coefficients for each channel as part of the design matrix (Figure S4) and PEB group level parameters gives estimates for first level DCM parameters.* |
