# Supplementary material for: Topographic Variation in Human Neurotransmitter Receptor Densities Explains Differences in Intracranial EEG Spectra
Source: Hum Brain Mapp. 2025 Oct 31;46(16):e70393. doi: 10.1002/hbm.70393 (PMC12576962; doi:10.1002/hbm.70393)
Supplement: Supplementary file 2 — Table S1: Neurotransmitter receptors. List of receptors included in the autoradiography study (Zilles and Palomero‐Gallagher 2017). [file HBM-46-e70393-s002.docx]

| \| **Neurotransmitter** \| **Receptor** \| **Primary Effect** \| **Mechanism** \| \| --- \| --- \| --- \| --- \| \| GABA \| GABA_A_ \| inhibitory \| ionotropic \| \|  \| GABA_A/BZ_ \| inhibitory \| ionotropic \| \|  \| GABA_B_ \| inhibitory \| metabotropic \| \| Glu (glutamate) \| Kainate \| excitatory \| ionotropic \| \|  \| AMPA \| excitatory \| ionotropic \| \|  \| NMDA \| excitatory \| ionotropic \| \| ACh (acetylcholine) \| M_1_ \| excitatory \| metabotropic \| \|  \| M_2_ \| inhibitory \| metabotropic \| \|  \| M_3_ \| excitatory \| metabotropic \| \|  \| α_4_β_2_ \| excitatory \| ionotropic \| \| NA (noradrenaline) \| α_1_ \| excitatory \| metabotropic \| \|  \| α_2_ \| inhibitory \| metabotropic \| \| DA (dopamine) \| D_1_ \| excitatory \| metabotropic \| \| 5-HT (serotonin) \| 5-HT_1A_ \| inhibitory \| metabotropic \| \|  \| 5-HT_2_ \| excitatory \| metabotropic \| |
| --- | --- | --- | --- | --- | --- | --- | --- | --- | --- | --- | --- | --- | --- | --- | --- | --- | --- | --- | --- | --- | --- | --- | --- | --- | --- | --- | --- | --- | --- | --- | --- | --- | --- | --- | --- | --- | --- | --- | --- | --- | --- | --- | --- | --- | --- | --- | --- | --- | --- | --- | --- | --- | --- | --- | --- | --- | --- | --- | --- | --- | --- | --- | --- | --- |
| ***Supplementary Table S1. Neurotransmitter receptors.*** *List of receptors included in the autoradiography study (Zilles & Palomero-Gallagher, 2017).* |
